# Osmotic-Adaptation Response of *sakA/hogA* Gene to Aflatoxin Biosynthesis, Morphology Development and Pathogenicity in *Aspergillus flavus*

**DOI:** 10.3390/toxins11010041

**Published:** 2019-01-14

**Authors:** Elisabeth Tumukunde, Ding Li, Ling Qin, Yu Li, Jiaojiao Shen, Shihua Wang, Jun Yuan

**Affiliations:** Key Laboratory of Pathogenic Fungi and Mycotoxins of Fujian Province, Key Laboratory of Biopesticide and Chemical Biology of the Ministry of Education and School of Life Sciences, Fujian Agriculture and Forestry University, Fuzhou 350002, China; elizawkb22@gmail.com (E.T.); echoliding@hotmail.com (D.L.); qlfafu@outlook.com (L.Q.); lisir9051@outlook.com (Y.L.); Sjjiao1108@outlook.com (J.S.)

**Keywords:** osmotic stress, aflatoxin, *AfsakA*, *Aspergillus flavus*, MAPK

## Abstract

*Aspergillu*s *flavus* is one of the fungi from the big family of *Aspergillus* genus and it is capable of colonizing a large number of seed/crops and living organisms such as animals and human beings. *SakA* (also called *hogA/hog1*) is an integral part of the mitogen activated protein kinase signal of the high osmolarity glycerol pathway. In this study, the *AfsakA* gene was deleted (*∆AfsakA*) then complemented (∆*AfsakA::AfsakA*) using homologous recombination and the osmotic stress was induced by 1.2 mol/L D-sorbital and 1.2 mol/L sodium chloride. The result showed that ∆*AfsakA* mutant caused a significant influence on conidial formation compared to wild-type and ∆*AfsakA::AfsakA* strains. It was also found that *AfsakA* responds to both the osmotic stress and the cell wall stress. In the absence of osmotic stress, ∆*AfsakA* mutant produced more sclerotia in contrast to other strains, whereas all strains failed to generate sclerotia under osmotic stress. Furthermore, the deletion of *AfsakA* resulted in the increase of Aflatoxin B_1_ production compared to other strains. The virulence assay on both maize kernel and peanut seeds showed that ∆*AfsakA* strain drastically produced more conidia and Aflatoxin B_1_ than wild-type and complementary strains. AfSakA-mCherry was located to the cytoplasm in the absence of osmotic stress, while it translocated to the nucleus upon exposure to the osmotic stimuli. This study provides new insights on the development and evaluation of aflatoxin biosynthesis and also provides better understanding on how to prevent *Aspergillus* infections which would be considered the first step towards the prevention of the seeds damages caused by *A. flavus*.

## 1. Introduction

The *A. flavus* is one of the fungi from the big family of *Aspergillu*s *genus* and this fungus is capable of colonizing a large number of seed/crops and living organisms such as animals and human beings [1,2]. *Aspergillus genus* is one of the most aflatoxigenic fungi ever identified worldwide [3,4]. Most of the highly toxic secondary metabolites (aflatoxins) are extensively produced by fungi from this group including *A. flavus*, *A. parasiticus* and *A. nomius*. If ingested by humans, these massively toxic secondary metabolites often cause acute and chronic toxicity which lead to serious health issues [2,5]. These findings were recently corroborated by Yu et al. [6] and later by Cleveland et al. [7] who further added that the aflatoxin biosynthesis pathway is one of the mostly understood pathways for fungal secondary metabolites. It is important to indicate that *A. flavus* has been known for a very long time not only as the major producer of mycotoxins but also as the foremost producer of the Aflatoxin B_1_ (AFB_1_) and B_2_, among which AFB_11_ is known to be the most poisonous [8,9].

As indicated earlier, *A. flavus* causes severe diseases to the seed/crops like peanuts, corn, rice, cotton, beans and wheat which lead to a significant loss of agriculture commodities. For example, in case *A. flavus* is highly inhaled or ingested by living organisms, this fungus can cause various fungal infections such as invasive and non-invasive human aspergillosis [10,11,12]. For more than 40 years, unlike *A. fumigatus*, *A. flavus* has been studied in animals’ standards even though they are hardly used [5]. Studies indicated that *A. flavus* is more virulent than any other *Aspergillus* species capable of producing toxins [5,13]. Therefore, a deep understanding on the signaling pathways that has an influence on the regulation of factors involved in this virulence is needed.

Mitogen activated protein kinases (MAPKs) are protein kinases which are present in eukaryotes. These proteins are not only capable of differentiating immune responses in mammalian cells but also capable of mediating differentiation of programs in their cells [14]. Their signaling pathways are necessary for the adaptation of the environmental changes and are controlled by phosphorylation cascades [15]. Their kinases-cascades are composed of three-component signal relay, that is, MAPKKK (MAP kinase kinase kinase), MAPKK (MAP kinase kinase) and MAPK (MAP kinase) and work in sequential activation. Once the cell receives different environmental stimuli, the former initiates an activation of a MAPK cascade. The MAPKKK first phosphorylates the MAPKK which phosphorylates MAPK on its turn [16,17,18]. Several studies propound that MAPKs are also referred to as SAPKs (stress activated protein kinases) and their pathways are able to respond to multiple stress signals [19,20,21]. The recent study by May et al. [22] and Nascimento et al. [13] reported that *A. fumigatus* contains 4 MAPK genes including *mpkA*, *mpkB*, *mpkC* and *sakA*/*hogA*. Their findings were corroborated by several other researchers who further reiterated that MAPK SakA plays an important role in stress response [14,23]. A study conducted by Jaimes-Arroyo et al. [24] also found that MAPK SakA is an integral components of a central multiple stress-signaling pathway that also regulates development in *A. nidulans*. Similar studies on other *Aspergillus* species revealed that *sakA* gene is a homologue to *hog1* gene of the *Saccharomyces cerevisiae*.

Of significance, *sakA* is also a part of MAPK signaling pathways in high osmolarity glycerol (HOG) pathway where it acts as an osmotic regulator [13,25]. Upon exposure to a hyperosmotic pressure, HOG pathway gets activated and causes the alteration in the expression of various genes in *S.cerevisiae* [15]. For example, upon exposure to a hyperosmotic pressure, genes which are essential for growth and their expression are controlled by HOG pathway, such as glycerol biosynthesis genes *GPD1* (glycerol-3-phosphate dehydrogenase) [26], *GPP1* and *GPP2* (glycerol-3-phosphotase) [27,28]. In a recent study, Duran et al. [15] corroborated what has been reported by several studies highlighting the influence of osmotic pressure on *Aspergillus* species as induced by solute concentrations, water content as well as water activity. In the case of *A. parasiticus*, moisture and substrate variation affected the development on corn kernels [29], whereas in *A. niger*, the hyperosmotic NaCl increased the secretion and production of glucose oxidase during fermentation [30].

Although *sakA* gene has been extensively studied in many different organisms, to our best knowledge, it has not been reported in *A. flavus*. Therefore, it was deemed necessary to investigate how MAPK AfSakA responds to different environmental stress. Therefore, in this study, we investigated the role of AfSakA in the presence and absence of osmotic stress and its subsequent response to conidia formation, sclerotia production, seeds pathogenicity as well as aflatoxin production.

## 2. Results

### 2.1. Identification and Analysis of sakA in A. flavus

**Figure 1:**
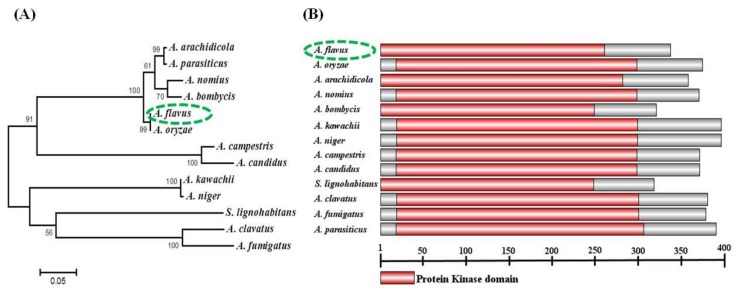
Bioinformatics analysis of SakA protein from 13 different fungi (**A**) Phylogenetic analysis of SakA protein from different fungi such as *A. flavus*, *A. oryzae*, *A. arachidicola*, *A. nomius*, *A. bombycis*, *A. kawachii*, *A. niger*, *A. campestris*, *A. candidus*, *S. lignohabitans (Sugiyamaella lignohabitans*), *A. clavatus* and *A. fumigatus*. The phylogenetic tree was created from different SakA aligned protein sequences from all the above listed fungi. Neighbor joining and bootstrap methods with 1000 replications were used to generate this phylogenetic tree. (**B**) Conserved domain analysis of the fungi. The gray color stands for the non-conserved part, the red color corresponds to protein kinase domain and the scale symbolizes the protein length.

Gene sequence of *AfsakA* was obtained from NCBI with the sequence ID AFLA-099500 and protein ID EED46287.1. MAP kinase AfSakA protein EED46287.1 was predicted to consist of 337-amino-acid. Then SakA protein sequences from other 12 different type of fungi namely *A. oryzae* (KDE75035), *A. arachidicola* (PIG85517) *A. parasiticus* (KJK67979), *A. nomius* (XP_015400855), *A. bombycis* (XP022385176), *A. kawachii* (GAA86222), *A. niger* (GAQ39538), *A. campestris* (XP_024694930), *A. candidus* (XP_024671978), *Sugiyamaella lignohabitans* (XP_018738467), *A. clavatus* (XP_001274144) and *A. fumigatus* (XP_753727.2) were also downloaded from NCBI. The recovered protein sequences were used in Molecular Evolutionary Genetic Analysis Version 7.0.26 (MEGA 7) software for the revolutionary relationship analysis between all these species. The results showed that AfSakA of *A. flavus* had the highest similarity to that of *A. oryzae* (96% identity, query cover 100%) (Figure 1A). Thus we analyzed SakA protein sequence in each fungi specie from Interpro (protein sequence analysis and classification) database. The identified protein kinase domain was constructed using a free software called Illustrator for Biological Sequences Version 1.0 (IBS 1.0) and all the 13 fungi share a highly conserved protein kinase domain (Figure 1B).

### 2.2. Generation of AfsakA Deletion and Complementation Mutant Strains

**Figure 2:**
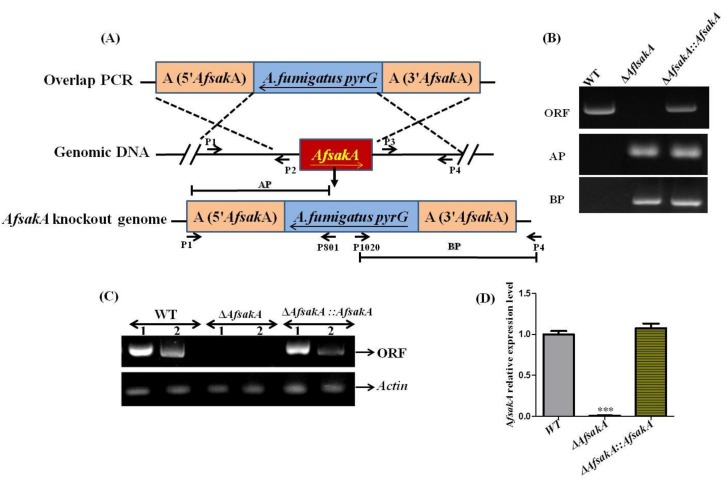
Schematic illustration of *AfsakA* deletion and complementation strains of *A. flavus*. (**A**) Homologous recombination technique was applied for the deletion of *AfsakA*. The fragments 5′ UTR (untranslated region) (*5′AfsakA*), *A. fumigatus pyrG* and 3′ UTR (*3′AfsakA*) were each amplified with primer pairs of P1/P2, *pyrG*-F/*pyrG*-R and P3/P4 and fused together with primer pairs P7/P8. The fusion PCR product was transferred into CA14 (*PyrG, Δku70*) in order to produce *ΔAfsakA* (*Δku70; pyrG; ΔAfsakA::pyrG*). (**B**) The results of the PCR analysis of *ΔAfsakA* and *∆AfsakA::AfsakA*. ORF (open reading frame), AP and BP fragments were amplified with a couple of primers P9/P10, P1/P801 and P4/P1020 respectively. (**C**) Verification of *AfsakA* deletion and complementary strains with reverse transcription-PCR. *Actin* gene was used as an endogenous control. Lane 1 used gDNA and lane 2 used cDNA as template. (**D**) qRT-PCR analysis of the expression level of *AfsakA* gene in *∆AfsakA* mutant, WT (wild-type) and *∆AfsakA::AfsakA* strains. The line bar in every column in (**D**) indicates standard errors of four replicates and the asterisks show significant difference between the knockout mutant and other strains (WT and complementation strain) (*** *p* ≤ 0.001).

To study the role of *AfsakA* gene in *A. flavus*, we constructed the knockout (∆*AfsakA*) and complementation mutant (∆*AfsakA::AfsakA*) strains by homologous recombination strategy (Figure 2A). To construct *AfsakA* deletion strain, the ORF of *AfsakA* gene was replaced by *pyrG* gene. Whereas, in order to confirm the role of *AfsakA* gene and to insure that *AfsakA* was fully functional, we created *AfsakA* complementation strain. Thus, we assembled complementation vector (pPTR-*AfsakA*) and transferred it into ∆*AfsakA* protoplast. The transformants were first proved by both PCR (Figure 2B) and the result showed that both AP and BP fragments were missing in the WT but present in ∆*AfsakA* and in *∆AfsakA::AfsakA* (Figure 2B), indicating that *A. fumigatus pyrG* had replaced ORF of *AfsakA*. The positive mutant strains were further confirmed by RT-PCR and the result in Figure 2C demonstrated that *AfsakA* gene was present both in the WT and in ∆*AfsakA::AfsakA* strains but it was absent in ∆*AfsakA*. Further results of qRT-PCR showed that *AfsakA* could not be expressed in ∆*AfsakA* (Figure 2D). Hence, all these results revealed that ∆*AfsakA* strain and ∆*AfsakA::AfsakA* strain were successfully constructed.

### 2.3. Effects of AfsakA Deletion on Osmotic Stress Response in A. flavus

**Figure 3:**
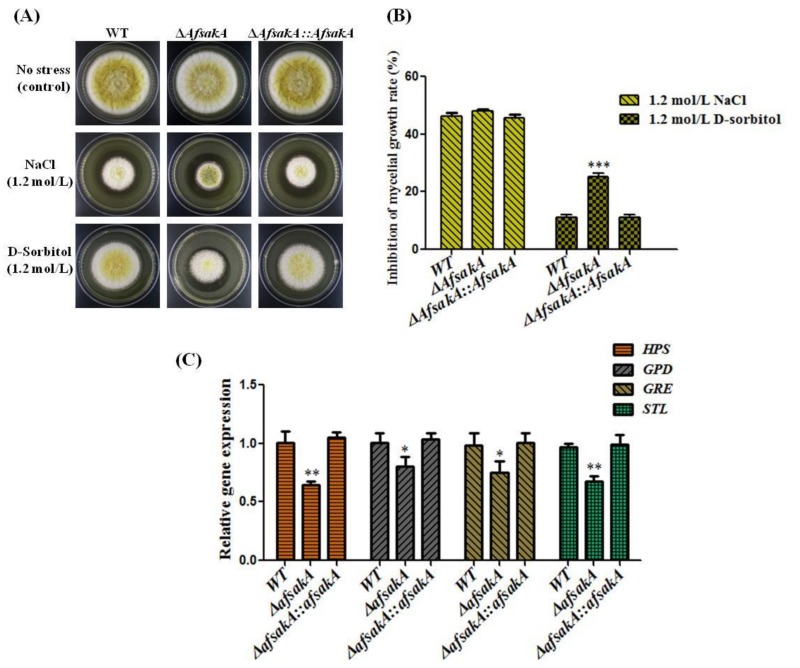
Phenotype and inhibition rate of WT (wild-type), ∆*AfsakA* and ∆*AfsakA::AfsakA* strains under osmotic stress induced by NaCl (sodium chloride) and D-sorbitol. (**A**) the morphology of WT, ∆*AfsakA* and ∆*AfsakA::AfsakA* strains under osmotic stress. (**B**) the mycelial growth inhibition of WT, ∆*AfsakA* and ∆*AfsakA::AfsakA* strains under osmotic stress. (**C**) qRT-PCR analysis of the expression level of the gene related with osmolality at 48 h without stress. The 2^−ΔΔCT^ method was used to calculate the expression levels of our target gene and *β-actin* gene was taken as a reference gene. The line bar in every column indicates the standard errors of the four replicates and the asterisks show significant difference level between WT and other strains (* *p* ≤ 0.05, ** *p* ≤ 0.01 and *** *p* ≤ 0.001).

To evaluate the potential functions of *AfsakA* under extracellular stimuli responses, we analyzed the sensitivity of WT, ∆*AfsakA* and ∆*AfsakA::AfsakA* to osmotic stress agents. To do this, 10^6^ conidia were inoculated onto YES agar media with and without the formerly described osmotic stress supplements. The cultures were then incubated for 4 days at 37 °C in dark condition. The results indicated that without the stress, WT and complementary strains grew a little better than ∆*AfsakA* mutant but not significant (Figure 3A). Subsequently we analyzed the expression level of *HPS*, *GPD*, *GRE* and *STL* genes which are related with osmolality and the result showed that the expression levels of all these 4 genes were significantly decreased in ∆*AfsakA* than that in WT and ∆*AfsakA::AfsakA* strains (Figure 3C). Then growth inhibition rate of WT, ∆*AfsakA* and ∆*AfsakA::AfsakA* strains under D-sorbitol and NaCl were also examined. Surprisingly, all the strains showed almost the same sensitivity to 1.2 mol/L NaCl, whereas ∆*AfsakA* mutant was more sensitive to hyperosmotic stress induced by 1.2 mol/L D-sorbitol than the WT and complementary strains strain (Figure 3A,B). These finding suggested that *AfsakA* is involved in responses to some hyperosmotic stress in *A. flavus*.

### 2.4. Effects of AfsakA Deletion on Conidial Production in A. flavus

**Figure 4:**
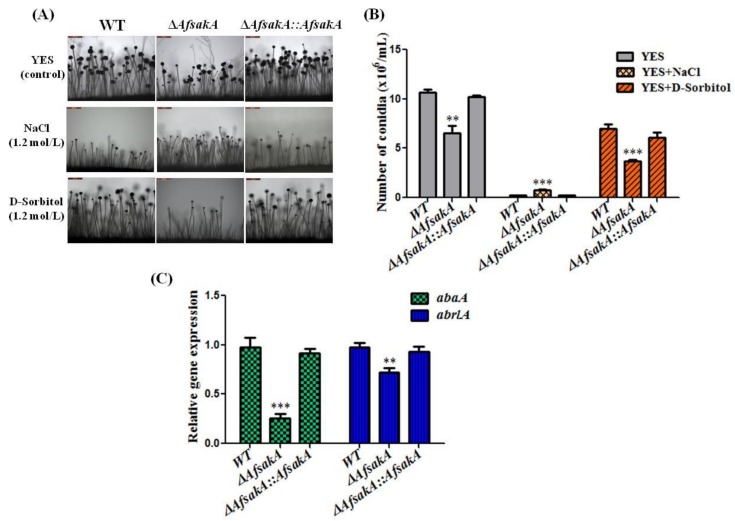
Conidiation analysis between WT(wild-type), ∆*AfsakA* and ∆*AfsakA::AfsakA* strains in the presence or absence of osmotic stress induced by NaCl (sodium chloride) and D-sorbitol. (**A**) Indicates the conidiophores under light microscope at 200× magnification. (**B**) Indicates the number of conidia produced by WT, ∆*AfsakA* and ∆*AfsakA::AfsakA*. (**C**) qRT-PCR analysis of the expression level of the gene related with conidia production in 48 h without osmotic stress agents. The line bar in each column in (**B**,**C**) shows standard errors of four replicates and the asterisks indicate significant difference between WT and other strains (** *p* ≤ 0.01 and *** *p* ≤ 0.001).

Conidia are the asexual spores which are produced by different type of fungi and their conidiation (asexual development) lead to mycotoxins production [31,32]. In this section, we analyzed the effect of ∆*AfsakA* on conidial and conidiophores formation under stress, in order to describe the function of *AfsakA* during asexual development. Microscopic examination showed that ∆*AfsakA* mutant produced less and short conidiophores than the WT and complementary ∆*AfsakA::AfsakA* strains in the absence of osmotic stress agents/supplements. In order to confirm the obtained variation in conidial formation, we examined the expression level of regulatory genes for conidial formation (*abaA* and *brlA*) during asexual development without stress. We found that the expression levels of these two genes were significantly decreased in ∆*AfsakA* compared to that in WT and complemented strains (Figure 4C). Under osmotic stress conditions induced by 1.2 mol/L D-sorbital or 1.2 mol/L NaCl, ∆*AfsakA* mutant indicated an extra reduction or increase of conidial formation respectively, compared to WT and ∆*AfsakA::AfsakA* (Figure 4A,B). These results suggest that *AfsakA* might be playing an important role in conidiation production and conidiophores formation.

### 2.5. Effects of AfsakA Deletion on Sclerotia Production in A. flavus

**Figure 5:**
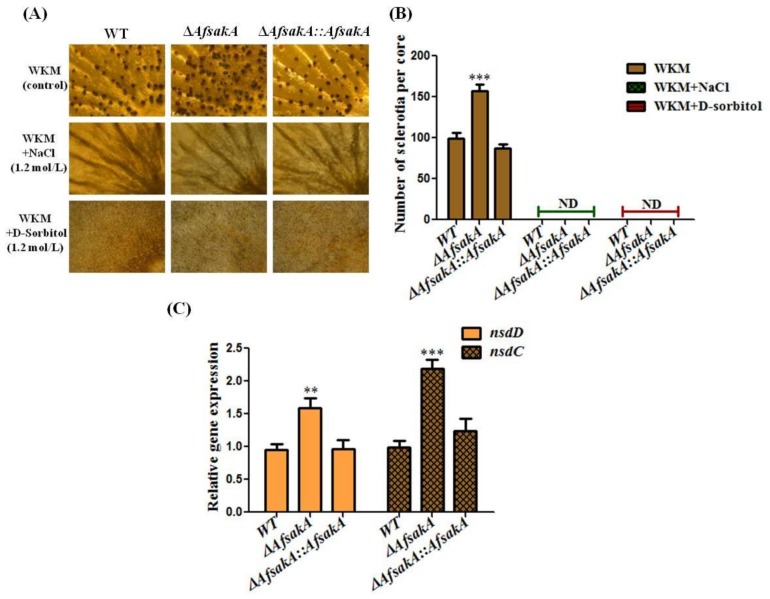
Sclerotia production analysis in the WT (wild-type), ∆*AfsakA* and ∆*AfsakA::AfsakA* strains. (**A**) presents the phenotype of WT, ∆*AfsakA* and ∆*AfsakA::AfsakA* to sclerotia formation. All the strains were grown in the dark for 7 days at 37 °C on WKM (wickerham) media with and without the indicated osmotic stress supplements. (**B**) reports the number of sclerotia produced from the four replicates. The abbreviation ND stands for “no detection” of sclerotia. (**C**) depictures the expression levels of *nsdC* and *nsdD* genes related with sclerotial development at 48 h without stress. The 2^−ΔΔCT^ process was used to calculate the expression levels of target gene which were normalized to *β-actin* as an endogenous gene. The asterisks show significant difference level when other strains were compared with WT (** *p* ≤ 0.01 and *** *p* ≤ 0.001) and the line bar in every column indicates standard errors of the four repeats.

It has been previously stated that *A. flavus* produces the sclerotia, which are known to be the sexual reproductive and survival structures that allow fungi to adjust to unsuitable environments [33] and some studies argued that sclerotia formation is inhibitory affected under osmotic stress [15,34]. In order to identify the role of *AfsakA* in sclerotia production, the strains were grown at 37 °C for 7 days on sclerotia-generating WKM medium in the presence/absence of osmotic stress agents. Results showed that sclerotia production in ∆*AfsakA* mutant was significantly increased on WKM without any osmotic stress agents in contrast to both WT and ∆*AfsakA::AfsakA* strains. Also, the expression levels of *nsdC* and *nsdD* genes related to sclerotial development was measured to confirmed the obtained results in sclerotia production, The results illustrated that the expression levels of both genes were higher in ∆*AfsakA* than that in ∆*AfsakA::AfsakA* and WT strains with no stress (Figure 5C). Of significance, WKM medium under osmotic stress could not produce sclerotia in all strains (Figure 5A,B). All these findings suggest that *AfsakA* could play a negative role in sclerotia production and osmotic stress might influence sclerotia formation in *A. flavus*.

### 2.6. AfsakA Plays a Negative Role in Regulating AFB_1_ Biosynthesis in A. flavus

**Figure 6:**
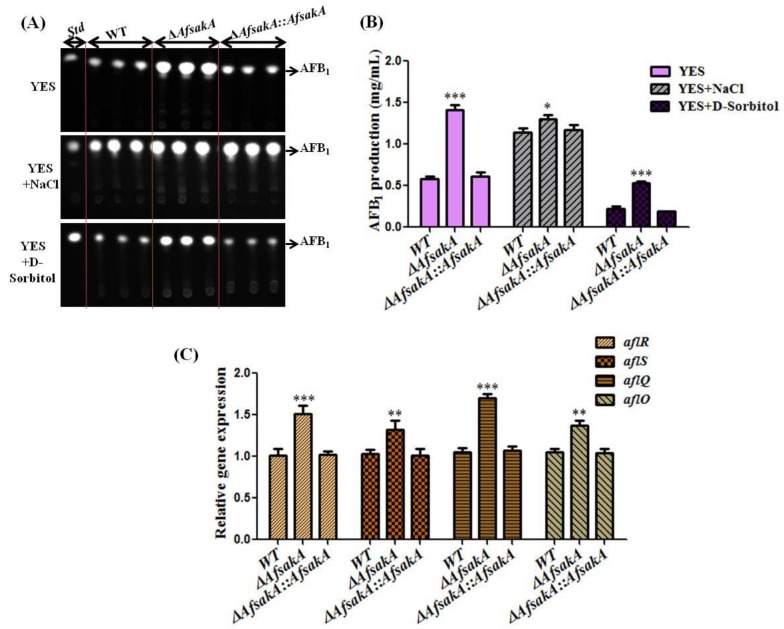
Aflatoxin B_1_ (AFB_1_) production analysis of WT, ∆AfsakA and ∆AfsakA::AfsakA strains. (**A**) AFB_1_ tested by tin layer chromatograph in all the strains after being grown in dark at 29 °C for 5 days on YES agar medium with or without osmotic stress supplements. The acronym Std correspond to the AFB_1_ standard. (**B**) the computation of AFB_1_ produced from TLC (Thin Layer Chromatograph) analysis in Figure 6A. (**C**) the qRT-PCR results of the expression level of aflR, aflS, aflQ and aflO genes related to the aflatoxin biosynthesis at 48 h in the absence of stress agents. The line bar in every column in (**B**,**C**) indicates the standard errors of the three replicates and the asterisks show significant difference level between WT and other strains (* *p* ≤ 0.05, ** *p* ≤ 0.01 and *** *p* ≤ 0.001).

To further investigate the role of *AfsakA* in biosynthesis of aflatoxin, WT, ∆*AfsakA* and ∆*AfsakA::AfsakA* strains were grown at 29 °C for 5 days on a YES agar medium with and without osmotic stress supplements and tested by TLC (Thin Layer Chromatograph) (Figure 6A). TLC results showed that in contrast to the WT and ∆*AfsakA::AfsakA*, ∆*AfsakA* mutant produced more AFB_1_ under YES and stressed medium (Figure 6A,B). Furthermore, we evaluated the expression levels of *aflR*, *aflS*, *aflQ* and *aflO* genes (genes related to aflatoxin biosynthesis) in these strains. We found that, without osmotic stress, the transcription levels of all 4 candidates were apparently enhanced for the disruption of *AfsakA* (Figure 6C). Taken together, these results indicate that *AfsakA* could reduce aflatoxin biosynthesis by suppressing gene expression of the aflatoxin cluster in *A. flavus*.

### 2.7. AfsakA Has an Influence on Virulence to Crop Seeds

**Figure 7:**
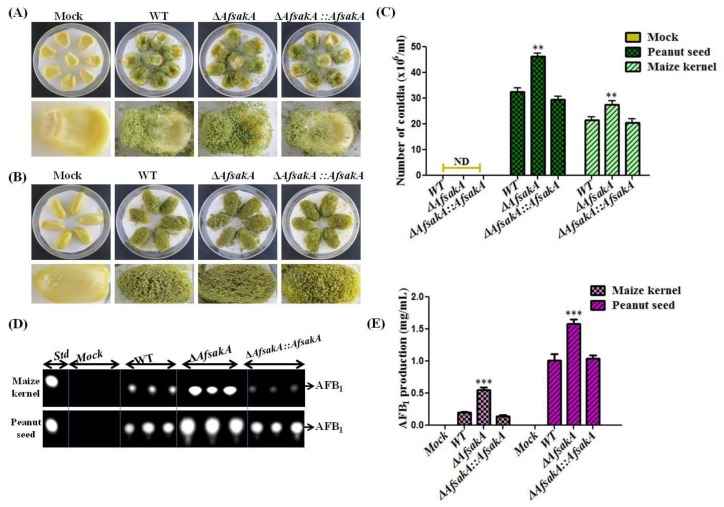
Effect of *AfsakA* deletion on fungal pathogenicity on peanut seeds and maize kernel. (**A**) Maize kernels were treated with ∆*AfsakA::AfsakA*, ∆*AfsakA*, WT strains and water (mock) and grown for 6 days at 29 °C in the dark. (**B**) Peanut seeds were treated with ∆*AfsakA::AfsakA*, ∆*AfsakA*, WT strains and water (mock) and grown in the dark for 6 days at 29 °C. (**C**) Quantitative evaluation of the amount of conidia produced on the infected crop seeds in every strain from (**A**,**B**). The abbreviation ND stands for “no detection.” (**D**) TLC analysis of Aflatoxin B_1_ (AFB_1_) produced by both the infected maize kernels and peanut seeds. (**E**) Relative amount of AFB_1_ produced in (**D**). The abbreviation Std corresponds to the AFB_1_ standard. The line bar in every column indicates standard errors of three replicates and the asterisks show significant difference level between WT and other strains (** *p* ≤ 0.01, *** *p* ≤ 0.001).

As indicated early, *A.flavus* is capable of colonizing a large number of seed crops. This characteristic was taken advantage of investigating the effect of *AfsakA* on pathogenicity. To do this, both maize kernels and peanut seeds were infected with ∆*AfsakA::AfsakA*, ∆*AfsakA* and WT strains and grown for 6 days at 29 °C in the dark. Seeds treated with water (mock inoculation) were used as a control to ensure they were free from any infection. We found that ∆*AfsakA* strain appeared to produce more dark green conidia than complementation and WT in both the maize kernels and peanut seeds (Figure 7A,B). We also calculated the amount of conidia produced by each strain on seed crops and found that *AfsakA* deletion mutant produced more conidia compare to other strains (Figure 7C). Consistent with the previously results for the strains grown in YES agar media, ∆*AfsakA* strain produced more AFB_1_ on the infected crop seeds than the WT and ∆*AfsakA::AfsakA* (Figure 7D,E). All these findings suggest that *AfsakA* may have an influence on virulence to crop seeds.

### 2.8. Subcellular Localization of AfSakA in A. flavus

**Figure 8:**
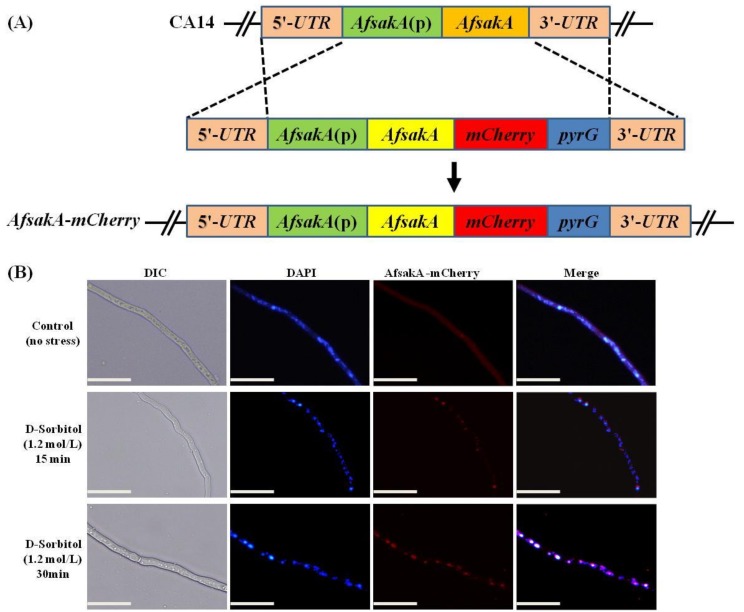
Subcellular allocation of AfSakA-mCherry fusion. (**A**) Gene replacement approach was used during construction of *AfsakA*(p)-*AfsakA*-*mCherry* strain. The UTR and *AfsakA*(p) abbreviations stand for “untranslated region” and “native promoter of *AfsakA,”* respectively. (**B**) Laser confocal scanning images of AfSakA-mCherry in vegetative mycelium. The AfSakA-mCherry strain was cultured for 16 h at 30 °C in YES liquid medium and cultured for 15, 30 min in the presence of 1.2 mol/L D-sorbitol. On the scale, 1 bar = 10 µm.

In order to identify subcellular location of the target AfSakA, mCherry tag was assembled at the C-terminal of AfSakA (*AfSakA*-*mCherry*) under the control of the native promoter (Figure 8A). The constructed *AfSakA*-*mCherry* vector was transferred into the protoplast of *A. flavus* CA14 strain. The *AfsakA*-*mCherry* showed the same phenotype as the WT (data not shown), implying that *mCherry* fusion did not affect the phenotype of *A. flavus*. The *AfsakA*-*mCherry* strain was grown overnight in YES liquid medium and osmotic stress agents were added to this medium while the control was left untreated. We found that, during the vegetative growth, the control showed a frail and diffused fluorescence signal in the cytoplasm of hyphae, whereas a strong fluorescence was observed in the hyphae’s nucleus on a medium with 1.2 mol/L NaCl after 15 and 30 min of incubation. (Figure 8B). These findings suggest that AfSakA is located in the cytoplasm in the absence of stress, while it translocated into the nucleus under osmotic stress.

### 2.9. Effects of AfsakA Deletion on Growth and Sensitivity to Stress

*AfsakA* involves in responses to osmotic and cell wall stress in many fungi. Therefore, we studied the effect of *AfsakA* under stress conditions in *A. flavus*. At the beginning of this study, a series of studies were conducted to identify the type of stress that has greater effects on growth and sensitivity following the deletion of *AfsakA*. The above was done by first analyzing the effects of *AfsakA* deletion on growth and sensitivity to different types of stresses using various stress stimuli. The former included genotoxic stress, cell wall stress, osmotic stress and oxidative stress. It was found that significant response was found under Cell wall and osmotic stress. 

**Figure 9:**
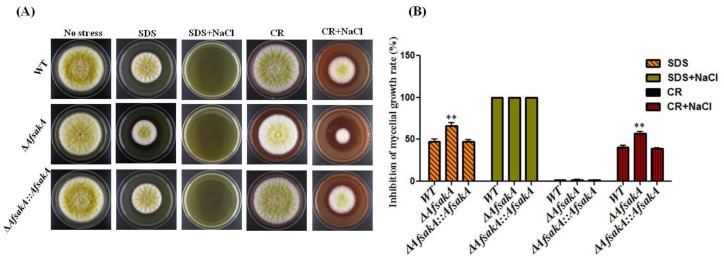
Phenotype and inhibition of growth rate of WT, ∆*AfsakA* and ∆*AfsakA::AfsakA* strains under cell wall stress only or together with osmotic stress. (**A**) All strains were grown on a control and on a YES agar media with various cell wall stress agents such as 100 µg/mL SDS (Sodium dodecyl sulfate) and 200 µg/mL CR (Congo Red) only or together with 1.2 mol/L NaCl. (**B**) Inhibition of mycelial growth rate. The line bar in column of the graph indicates standard errors of all three replicates and the asterisks demonstrate significant difference levels between WT and other strains (** *p* ≤ 0.01).

Consistent with the aforementioned, we then decided to find out what might happen once the cell wall stress alone and/or osmotic stress is applied. As such, we investigated the sensitivity of WT, ∆*AfsakA* and ∆*AfsakA::AfsakA* strains under cell wall stress and/or osmotic stress. All strains were spotted on YES agar medium in the absence of stress stimuli (control) and with cell wall damaging agents (SDS, CR) with and without osmotic stress agent (NaCl). All the cultures were grown in the dark for 4 d at 37 °C and photographed (Figure 9A). Then, we measured the diameter of each colony and calculated the growth rate inhibition (Figure 9B). We observed that on media with SDS and CR+NaCl, the ∆*AfsakA* mutant was more sensitive than the WT and ∆*AfsakA::AfsakA*. However, on media containing CR, the ∆*AfsakA* mutant showed slightly relative levels of stress tolerance in contrast to WT and ∆*AfsakA::AfsakA*. Interestingly, medium supplemented with SDS+NaCl completely inhibited growth of all strains. The aforementioned findings suggest that *AfsakA* responds to all simultaneously induced stresses (cell wall stress + osmotic stress) and cell wall stress stability in *A. flavus*.

## 3. Discussion

It has been demonstrated that MAPK signaling pathway plays an important role in eukaryotic organisms and cascades of MAPK involves in regulating the apoptosis, growth, expression of the genes, hyperosmoregulation, cell division, cell endurance and ascospore development [17,19]. Similarly, in both *A. fumigatus* and *A. nidulans*, *sakA* gene intervenes in MAPK signaling cascades in HOG pathway as an osmotic regulator. *SakA* gene is also a homologue of *hog1* within the *S. cerevisiae* [13,14,19] and acts as an essential response to numerous types of stress [13]. In the present study, our main interest was to understand how MAPK *AfsakA* gene responds to osmotic stress signals in *A. flavus*. During the identification process of our target gene in *A. flavus*, we realized that *sakA* gene was conserved among all *Aspergillus* species. The sequence alignments of all the analyzed fungal species revealed that *sakA* has high similarity as they all share a highly conserved protein kinase domain. All these results confirmed that *sakA* is conserved in MAP kinase/SAPKs family, which is in consistence with earlier studies that *sakA* gene was found to encode a part of MAPK gene family in both *A. nidulans* [19] and *P. marneffei* [20].

The function of *AfsakA* in responses to osmotic stress was examined. ∆*AfsakA* proved to be more sensitive to 1.2 mol/L D-Sorbitol than WT and ∆*AfsakA::AfsakA* strains and our results also demonstrated that growth rate in ∆*AfsakA* could be related to the expression of the *HPS*, *GPD*, *GRE* and *STL* genes which are related with osmolarity. Surprisingly, when using 1.2 mol/L NaCl, no growth rate differences were detected in all the strains. As a result, we suspect that *AfsakA* involves in multiple mechanisms that contribute to osmotic regulation. The above was corroborated by Xue et al. [23] who reiterated that *A.fumigatus sakA* deletion affect growth and conidia germination at a high osmolarity (1 mol/L NaCl). The current results were also supported by the findings of Nimmanee et al. [20] and Hagiwara et al. [31] who previously reported that *A. fumigatus sakA* gene contributes to the regulation of hyperosmotic stress.

Conidiophores are known as the multicellular structures which produces conidia during the vegetative growth under favorable conditions and in a particular period of time [32]. In the present study, we evaluated the role of *AfsakA* gene in asexual development (conidiation) and we found that ∆*AfsakA* mutant produced less conidia than other strains in the absence of environmental stress. In the presence of extracellular stress induced by 1.2 mol/L NaCl, ∆*AfsakA* exhibited a significant increase in conidial formation, whereas with 1.2 mol/L D-sorbitol, ∆*AfsakA* illustrated a decrease in conidial formation in contrast to WT and ∆*AfsakA::AfsakA* strains. We concurrently found that without stress the expression levels of *abaA* and *brlA* genes which are related with conidia production were significantly down-regulated in ∆*AfsakA* compared to that in both WT and complemented strains. Previous studies indicated that an increased amount of sorbitol increases the conidia production in *A. nidulans* [15,35]. Mert et al. [36] and Duran et al. [15] also indicated that a decrease in conidia production was observed when *A. flavus* was cultured on a medium with a high amount of NaCl. These results taken together indicate that *AfsakA* might play an essential role in asexual development more especially in the survival of conidia and conidiophores under harsh conditions. This corroborates the findings in the earlier study by Kawasaki et al. [19] which reported that *A. nidulans sakA* plays a role in the asexual development where it regulates the genes that are involve in sustention of conidia and their persistence.

Sclerotia are the sexual reproductive and survival structures which are produced by different fungi which helps fungi to adapt to unsuitable environmental conditions [33]. Our results indicated that *A. flavus* produced sclerotia in the absence of stress and the deletion of *AfsakA* increased sclerotia production in contrast to other strains. This might be due to inactivation of *AfsakA* affected other signal pathway that are involved in normalization of sexual development in *A. flavus*. Further, the qRT-PCR results of the expression level of *nsdC* and *nsdD* genes related to sclerotial development indicated that the expression levels of both genes were significantly up-regulated in ∆*AfsakA* than that in ∆*AfsakA::AfsakA* and WT. Putting all these results together, we can confirm that *AfsakA* regulates the sexual development of *A. flavus* and the hyperosmotic stress may have been preventing, delaying or inhibiting sclerotia formation. Our results showed that under osmotic stress, sclerotia could not be produced in all strains after 7 days of incubation at 37 °C. Other studies also confirmed that salt stress have inhibitory effect on sclerotia production in various fungal species viz. *Sclerotium rolfsii*, *Sclerotinia sclerotiorum* and *Rhizoctonia solani* [15,34]. Moreover, stressed fungi with different types of salts produced fewer sclerotia in contrast to the unaltered control [34] after 15 d of incubation. In 1999, Ramos et al. [37] also reported that *A. ochraceus* treated with different levels of water activity exhibits different levels of sclerotia formation. They went on and indicated that sclerotia were freely produced in *A. ochraceus* treated with more than 0.99a_w_, while only initials were formed for *A. ochraceus* treated with 0.975a_w_ and no sclerotia at all were produced for *A. ochraceus* treated less than 0.975a_w_.

The characterization of the function of *AfsakA* in aflatoxin production was also performed. Obtained data proved that *∆AfsakA* considerably increased the production of AFB_1_ compared to WT and *∆AfsakA::AfsakA* when the agar medium was induced by hyperosmotic stress agents. This finding is consistent with the observed increase of the transcription levels of *aflR*, *aflS*, *aflQ* and *aflO* genes which are related to aflatoxin biosynthesis. Therefore, we can conclude that *sakA* gene acts by negatively regulating the aflatoxin formation while the osmotic stress affects the biosynthesis of aflatoxin in *A. flavus*. However, the details on the function of *sakA* gene in AFB_1_ biosynthesis under hyperosmotic are still unknown in other world of fungi.

In a recent study, Nascimento et al. confirmed that *sakA* and *mpkC* collaborate during *A. fumigatus* virulence in neutropenic mice [13]. An earlier study by Rementeria et al. [38] indicated that not only *sakA* involved in the cellular homeostasis regulation but also participates in a coordinated response to H_2_O_2_. Moreover, it has been demonstrated that *sakA* homologue, that is, *hog1* gene, has a major function in virulence and stress response in *Cryptococcus neoformans* [39]. In the present study, we examined the role of *AfsakA* in pathogenicity by infecting both the maize kernels and peanuts seeds. Results revealed that ∆*AfsakA* strain on the infected crop seeds appeared to produce a higher number of dark-green conidia than the WT and complemented strains in *A. flavus* and *∆AfsakA* increased the production of AFB_1_ on the infected crop seeds than the WT and *∆AfsakA::AfsakA.* The above findings suggest that *AfsakA* has an influence on virulence and pathogenicity.

Here, we also addressed the effect of *AfsakA* on sensitivity of *A. flavus* to cell wall stress and the simultaneous induction of both cell wall and osmotic stresses. We discovered that the growth inhibition of *∆AfsakA* mutant was greater on media with SDS and CR+NaCl compared with WT and *∆AfsakA::AfsakA*. However, all strains were unable to growth in the presence of SDS+NaCl. Our results suggested that *AfsakA* gene has an influence on these two simultaneous induced stresses and on cell wall integrity. Our results was reliable to the former studies on *A. fumigatus* which showed that *sakA* plays the important functions in responses to various stresses such as osmotic stress, heat shock, oxidative stress and cell wall damage [13]. Nimmanee et al. [17] reported that *S.cerevisiae Hog1* protein responds to UV, heat, oxidative stress, heavy metal and cell wall interfering agents. These studies also mentioned that Hog1 protein has an important role in the transmission process of osmotic pressure signals. So far, no report was found on the response of *sakA* under simultaneously induced stress, that is, cell wall stress together with osmotic stress.

Alves de Castro et al. [40] found that SakA was translocated to the nucleus of *A. fumigatus* after 30 min of exposure to osmotic stimuli. Similarly, Nascimento et al. [13] noticed a fast migration of *A. fumigatus* SakA to the nucleus after 10 min of exposure to osmotic stress. This was in consistence with our result which showed that AfsakA was located into cytoplasm of hypae in the absence of osmotic stress and migrated to the nuclei of the hyphae upon the exposure to the osmotic stress. Our results were also supported by the findings by Lara-Rojas et al. [41] which indicated that in the absence of an induced stress on hyphae, *A. nidulans* SakA was not located in the nucleus.

## 4. Conclusions

In conclusion, *AfsakA* gene was deleted (∆*AfsakA*) and complemented (∆*AfsakA::AfsakA*) using homologous recombination. We found that *AfsakA* plays an essential role in the production of sclerotia, conidiophores and conidia, as well as mycelia growth, AFB_1_ production and virulence in *A. flavus*. In addition, *AfsakA* plays a tremendous role in the regulation of both the osmotic and cell wall stresses in *A. flavus*. It was also found that AfSakA is located in the cytoplasm in the absence of osmotic stress and translocates into the nucleus upon exposure to hyperosmotic stress. It is believed that this research provides a more reliable and practical insights on how to control *A. flavus* infections based on the full understanding of *AfsakA* gene functions. Moreover, our findings present the vital information that helps us to understand how to prevent the aflatoxin biosynthesis. Consistent with the aforementioned, we found that it is possible to mitigate damages caused by *A. flavus* to the agriculture commodities and to control the invasive fungal infections in human and animals by storing both the harvested crops and food commodities in conditions different to those that favor the production of AFB_1_ (i.e., the latter being in the dark at 29 °C). Furthermore, we found that *AfsakA* negatively regulated the biosynthesis of AFB_1_. In the future work, we could find the inducers of *AfsakA* to inhibit the toxin production in the crops such as peanut and maize.

## 5. Materials and Methods

### 5.1. Fungal Strains and Culture Conditions

All *A. flavus* strains used in this study were described in Table 1. The plasmids DNA were extracted from *E. coli* strain. YES (Yeast Extract Sucrose) media [42] supplemented with or without 1.2 mol/L NaCl, 1.2 mol/L D-sorbitol were used to evaluate the growth rate, conidial development and AFB_1_ production. Similarly, YES media supplemented with or without 100 µg/mL SDS (Sodium dodecyl sulfate) and 200 µg/mL Congo Red were used to analyze the influence of cell wall stress after *AfsakA* deletion. Also, a wickerham medium (WKM) [43] complemented with/without osmotic stress agents was utilized to determine sclerotia production. The wild-type (WT), knockout and complementation mutant strains were cultured and grown in dark condition at the 37 °C and 29 °C which are the optimum temperatures for *A. flavus* growth and AFB_1_production respectively. Every strain was cultured and grown at least on four replicate plates and every experiment was repeated three times.

### 5.2. Sequence and Phylogenetic Tree Analysis

*AfsakA* gene sequence with the accession number AFLA-099500 and protein sequence with query ID EED46287.1 were obtained from the National Center For Biotechnology Information Database (NCBI). Protein sequences of other *sakA* orthologs were also downloaded from NCBI using blast algorithm with AfSakA protein sequence as inquiry. Protein kinase domains were constructed by using free software called Illustrator for Biological Sequences Version 1.0 (IBS1.0). Molecular Evolutionary Genetic Analysis Version 7.0.26 (MEGA 7) software was used to align sequences (ClustalW toolbox) and created the phylogenetic tree.

### 5.3. Construction of AfsakA Deletion and Complementation Strains

The deletion mutant Δ*AfsakA* was obtained by using homologous recombination method [42,45]. Standard PCR and *A. flavus* genomic DNA extraction were also carried out as formerly described [46]. Both 1307 bp upstream and 1096 bp downstream sequences of *AfsakA* gene were obtained from https://www.ncbi.nlm.nih.gov/ and the primers used in this study were listed in Table 2. Overlap PCR was carried out to join them and form a knockout cassette as A-*pyrG*-B. *A. flavus* CA14 were used as a starting strain during the preparation of protoplast [47] and the knockout cassette transformation was conducted and successively verified as previously described [44,47]. In order to obtain complementation strain *∆AfsakA::AfsakA*, we followed the method described by Yao et al. [48] and transformants were verified by PCR and qRT-PCR. The primers used for qRT-PCR were listed in Table 3.

### 5.4. Stress Assay

A YES agar medium supplemented with/without osmotic stress mediators (1.2 mol/L NaCl, 1.2 mol/L D-Sorbitol) and cell wall stress agents (100 µg/mL SDS, 200 µg/mL Congo Red) were inoculated with WT, knockout and complementary *A. flavus* strains. Four plates were used for each strain and incubated for 4 days in dark conditions at 37 °C (optimum growth conditions). The mycelium diameter was then measured and the growth inhibition rate was subsequently calculated. The following formula was used for this calculation: Growth rate inhibition = ((diameter of the unstressed strain—diameter of the stressed strain)/diameter of the unstressed strain) × 100.

### 5.5. Morphological Analysis

To determine the amount and quality of sclerotia, conidiophores and conidia, all the *A. flavus* strains were grown, photographed, measured and observed under microscope. To evaluate conidial formation, YES solid media in the presence or absence of osmotic stress agents were poured onto plates (15 mL per plate). After 4 days, conidia were harvested and counted as previously described [49,50]. In order to evaluate the amount of conidiophores produced, YES solid media with and without stress was also used and the strain cultures and mycelia were grown in the dark at 37 °C for 2 days. The hyphae were cut and added to a glass slide and observed through light microscope the following day. Determination of sclerotial formation was carried out as a former described method [51] with minor modification. In short, all strains were inoculated in WKM agar media supplemented with/without osmotic stress compounds as mentioned above, then grown in the dark for 7 days at 37 °C. Afterward, conidia were washed with 75% ethanol to allow visualization and counting of sclerotia.

### 5.6. Determination of AFB_1_ Production

*A. flavus* WT, Δ*AfsakA* and Δ*AfsakA::AfsakA* strains were inoculated onto a petri dish with 15 mL of YES agar media in the presence/absence of the aforementioned osmotic pressure agents. AFB_1_ was analyzed as previous method [47,52,53] with slight modifications. To produce AFB_1_, cultures were incubated for 5 days under the optimum condition (29 °C in dark conditions). Chloroform was used to extract the AFB_1_ and the extracts were spotted on TLC (Thin layer chromatography plate). The plate was added into a developing chamber containing a developing solvent of chloroform and acetone (9:1 *v*/*v*) and results were viewed under ultra violet (UV) light at 365 nm. Gene Tools image analysis system software version 4.03.05.0 was used for the quantification of AFB_1_.

### 5.7. Localization of AfsakA gene in A. flavus

*AfsakA-mCherry* strain was prepared by using a modified published methods [54,55] and the used primers were listed in Table 2. Then, the target gene *AfsakA* was tagged with *mCherry* for the construction of *AfsakA-mCherry* vector through the PCR amplification of four different fragments. *AfsakA* ORF fragment, *mCherry* fragment, selection marker *pyrG* and the downstream region of *AfsakA* were amplified and fused together by the overlap PCR. The mycelium of positive construct were cultured onto YES liquid medium and grown at 30 °C overnight in the shaker. Different osmotic stress agents were added. Then, mycelia were washed with PBS (Phosphate buffer saline) and stained with 1 µg/mL DAPI (4,6-diamidino-2-phenylindole) to allow the observation of the nucleus. Then, *AfsakA* gene location was checked in the hyphae for all the stress conditions and at different time interval, using laser confocal scanning microscope.

### 5.8. Pathogenicity Test

Peanut and maize seeds were used for pathogenicity assay according to the previously illustrated procedure [56,57]. In brief, the maize seed and peanuts cotyledons were inoculated with *A. flavus* 10^5^ conidia/mL and mock inoculation was used as a control. The seeds were placed in a petri dish (4 replicates for each sample) with a piece of moisture filter paper and incubated for 6 days at 29 °C in a dark condition. Peanuts and maize seeds were collected and placed into 50 mL centrifuge tubes containing 0.05% tween 80 and 15 mL of sterilized water and vortexed for 1 min. For conidia counting, 500 µL aliquot of conidia was diluted and counted haemocytometrically. Aflatoxin was extracted as previously described method by Yuan et al. [58]. The extracted samples were spotted on TLC plate and viewed under ultra violet (UV) light at 365 nm. Gene Tools image analysis system software version 4.03.05.0 was used for the quantification of AFB_1_.

### 5.9. Reverse Transcriptase Polymerase Chain Reaction (RT-PCR) and Quantitative Real Time Polymerase Chain Reaction (qRT-PCR)

qRT-PCR were carried out following the descriptions and guidelines that were previously described [53,55] with minor modifications. Consistent with the aforementioned, *A. flavus* wild-type and mutant strains were inoculated into YES agar media and grown in the dark for 2 days at 37 °C. Their mycelia were then collected and strongly grounded in liquid nitrogen to break down the cell wall material and get access to RNA molecules isolation. The total RNA was extracted from 100mg smashed mycelia for every sample using Total RNA Extraction kit (Promega, Madison, WI, USA). The first strand cDNA for each sample was synthesized from the extracted total RNA using revertAid first strand cDNA synthesis kit (ThermoFisher scientific, Waltham, MA, USA). To analyze qRT-PCR 2xSYBR Green mix kit (Takara Dalian China), a pair of primers (Table 3) for target genes were used and amplified with ThermoFisher scientific Real-time PCR system (PikoReal 96 Real-Time PCR system/from Vantaa, Finland). The valuation of relative transcript level of every target gene was done by the 2^−ΔΔCT^ method [59] and *A. flavus actin* gene was used as a control. The qPCR test was carried out with technological quadruplet for wild-type, *ΔAfsakA* and *ΔAfsakA::AfsakA* and the experiments was repeated at least in triplicate.

### 5.10. Statistical Analysis

GraphPad Prism Version 5.01 software was used for significance and statistical analysis and every data were presented as ± standard deviation (SD). Afterward, Colum analysis with one way ANOVA were used to find out the presence of statistical differences between the grouped data of at least replicates three values of *A. flavus* wild-type, *ΔafsakA and ΔafsakA::afsakA*. Also, for determining the significance difference among the groups, Dunnett’s multiple comparisons test method was used and every column group was compared with control column (WT). Asterisks were used to represent significant different whereby * represents significant different *p* ≤ 0.05, ** stands for significant different *p* ≤ 0.01 and *** correspond to significant different *p* ≤ 0.001.

## Figures and Tables

**Table 1:** *A. flavus* strains used in this study.

Strain Name	Related Genotype	Source
CA14	Δ*ku70*; *niaD*-; Δ*pyrG*	Chang et al. [44]
Wild-type	Δ*ku70*; *niaD*-; Δ*pyrG::pyrG*	This study
Δ*AfsakA*	Δ*ku70*; *ΔAfsakA::pyrG*	This study
Δ*AfsakA*::*AfsakA*	Δ*ku70*; Δ*AfsakA*::*pyrG*; *AfsakA* (p)::*sakA*::*ptrA*	This study
*AfsakA*-*mCherry*	Δ*ku70*; *AfsakA* (p)::*mCherry::AfsakA*::*pyrG*.	This study

**Table 2:** Primers used for gene deletion, complementary and localization.

Primers	Sequence (5′-3′)	Application
P1	TGTTATAGGGACGCTCTG	*AfsakA* deletion
P2	CGAACGTAGTACCCAAGAT	
P3	CGGAGCATTGTCCTACAT	
P4	GAGCGAATTACTGTTTGAGT	
P7	CATGTCGGGACGAGTTTG	
P8	GAAGTGGTTTACGGTGGT	
P9	ATACGCCTGCAACGCTAA	
P10	ATCCGCCTGGAGAAAGTC	
*pyrG*-F	GCCTCAAACAATGCTCTTCACCC	*pyrG* amplification
*pyrG*-R	GTCTGAGAGGAGGCACTGATGC	
P801	CAGGAGTTCTCGGGTTGTCG	*AfsakA* mutant verification
P1080	ATCGGCAATACCGTCCAGAAGC	
AfSakA-F	TGTTATAGGGACGCTCTG	*AfsakA* complementation
AfsakA-R	TCATATATCCGCCTGGAGAAAG	
*mCherry*/F	ATGGTGAGCAAGGGCGAG	*AfsakA- mCherry* tag construction
*mCherry*/R	GGGTGAAGAGCATTGTTTGAGGCCTACTTGTACAGCTCGTCCAT	
*pyrG*-R/R	GCCTCAAACAATGCTCTTCACCC	
*pyrG*-R/F	AGTTGGTACGAAACAGATCAGTCTGAGAGGAGGCACTGATGC	
*AfsakA*-mCherry/F	TCGGTTTGGGTGCGTTTG	
*AfsakA*-mCherry/R	CTCGCCCTTGCTCACCATGACTAGTTTGTAAAGTTTACTTTGGACTAT	
*AfsakA*-B/F	GTCTGAGAGGAGGCACTGATGCTGATCTGTTTCGTACCAACT	
*AfsakA*-B/R	TGAGACCGCCGTCCTAAC	
*AfsakA*-O/F	GGTTTGGGTGCGTTTG	
*AfsakA*-O/R	CCCGAATTTATTGTAGCG	

**Table 3:** Primers used for qRT-PCR.

Primers	Sequence (5′-3′)	Application
Q-*AfsakA*-F	CCCACTACCAAAGGCACTC	*AfsakA* detection
Q-*AfsakA*-R	GGCATCATTGAACGACCAG	
*AflO*-F	GATTGGGATGTGGTCATGCGATT	*AflO* qRT-PCR
*AflO*-R	GCCTGGGTCCGAAGAATGC	
*AflQ*-F	GTCGCATATGCCCCGGTCGG	*AflQ* qRT-PCR
*AflQ*-R	GGCAACCAGTCGGGTTCCGG	
*AflR*-F	AAAGCACCCTGTCTTCCCTAAC	*AflR* qRT-PCR
*AflR*-R	GAAGAGGTGGGTCAGTGTTTGTAG	
*AflS*-F	GCTCAGACTGACCGCCGCTC	*AflR* qRT-PCR
*AflS*-R	GCTCAGACTGACCGCCGCTC	
*NsdC*-F	GCCAGACTTGCCAATCAC	*NsdC* qRT-PCR
*NsdC*-R	CATCCACCTTGCCCTTTA	
*NsdD*-F	GGACTTGCGGGTCGTGCTA	*NsdD* qRT-PCR
*NsdD*-R	AGAACGCTGGGTCTGGTGC	
*abaA*-F	TCTTCGGTTGATGGATGATTTC	*abaA* qRT-PCR
*abaA*-R	CCGTTGGGAGGCTGGGT	
*brlA*-F	GCCTCCAGCGTCAACCTTC	*brlA* qRT-PCR
*brlA*-R	TCTCTTCAAATGCTCTTGCCTC	
*HSP*-F	CCGGCATACTATGTCTCGTCT	*HSP* qRT-PCR
*HSP*-R	TAGGGCCTTCGTCGAACA	
*GPD*-F	TGTCTCGGTGGTGTCCCTAT	*GPD* qRT-PCR
*GPD*-R	ACCATGGCTGATGGAAGACT	
*GRE*-F	GCGTATCGTCGTTACCTCATC	*GRE* qRT-PCR
*GRE*-R	CCTTCTCCTTTACCTCCTCGAT	
*STL*-F	CGTTTACCACGACCAGAGC	*STL* qRT-PCR
*STL*-R	AAGCTCAAGCCATGTGCAG	
*Actin*-F	ACGGTGTCGTCACAAACTGG	*Actin* qRT-PCR
*Actin*-R	CGGTTGGACTTAGGGTTGATAG	
